# Pros and cons of radical cystectomy in the treatment of T1G3 bladder cancer

**DOI:** 10.4103/0970-1591.35760

**Published:** 2008

**Authors:** Piyus K. Agarwal, Ashish M. Kamat

**Affiliations:** Department of Urology, the University of Texas M. D. Anderson Cancer Center, 1515 Holcombe Blvd, Unit #1373, Houston, TX 77030, USA

**Keywords:** Bladder cancer, cystectomy, intravesical therapy, nonmuscle-invasive, T1G3, transitional cell carcinoma, urothelial carcinoma

## Abstract

The management of T1G3 (or high-grade T1) bladder cancer poses a challenging clinical dilemma to the urologist. There are good data supporting bladder conservative therapy with repeat transurethral resection and administration of Bacille Calmette-Guérin (BCG) intravesical therapy but this must include maintenance regimens since only maintenance BCG has been shown to decrease tumor recurrence and progression. Concern over worse survival with a delay in definitive therapy has prompted many to recommend immediate cystectomy for T1G3 disease. Ultimately, the decision needs to be individualized and although certain pathologic factors (T1b disease, early recurrence or progression within three months of BCG therapy, lymphovascular invasion and variant histology) are prognostic of progressive disease, no definitive risk factors for tumor progression have been identified.

## INTRODUCTION

In 2002, bladder cancer of all types accounted for 145,009 deaths worldwide.[1] In the United States, bladder cancer accounted for 13,060 deaths in 2006. It is the fourth most common malignancy in men and the ninth most common malignancy in women with 44,690 men and 16,730 women being diagnosed in 2006.[2] Transitional cell carcinoma (TCC) is identified in greater than 90% of the cases.[3] The prognosis and treatment of TCC of the bladder is guided by the depth of tumor invasion into the bladder wall, tumor grade and the presence of any regional or distant metastases. Patients who present with muscle-invasive disease or distant metastases are usually managed by radical cystectomy or chemotherapy with or without radical cystectomy, respectively. Most bladder cancer patients (60-70%), however, present with nonmuscle-invasive disease. The management for these patients entails transurethral resection (TUR) with or without adjuvant intravesical therapy. Despite adequate therapy, however, 60-70% of these lesions will recur and 10-20% will progress to muscle-invasive disease, thereby requiring a radical cystectomy.[4] The challenge to the urologist is to identify those patients likely to develop progression of disease initially so that they can be treated aggressively upfront.

The decision to proceed with a radical cystectomy in nonmuscle-invasive disease is not always straightforward and is not to be taken lightly. Certainly, recalcitrant nonmuscle-invasive disease and Bacille Calmette-Guérin (BCG) refractory disease may ultimately require bladder removal. Other lesions such as TaG1 are obvious candidates for transurethral resection, possible intravesical therapy and continual surveillance. The T1G3 lesion, however, presents a dilemma to the treating urologist. “To remove or not to remove” the bladder? - that is the question. Unfortunately, no randomized trials comparing the results of immediate versus delayed cystectomy exist for T1G3 disease and so we must extrapolate from existing data. This review will attempt to review the salient points a urologist should evaluate in making the difficult decision of when to perform a radical cystectomy in these select patients.

## CASE AGAINST CYSTECTOMY

### Accurate staging of T1G3 disease allows consideration of conservative management

In order to engage in conservative management, one must be confident of the stage and grade of the tumor. This presupposes that a single TUR is adequate to make this diagnosis. However, Herr has elegantly demonstrated that repeat TUR of nonmuscle-invasive disease two to six weeks after initial TUR can up-stage 29% of tumors and change disease management in up to 33%. This should be performed even in patients who have muscle present in the initial specimen since, of 35 patients with T1 disease and muscle present on initial TUR, five (14%) were upstaged to T2 disease on repeat TUR.[5] Therefore, a repeat TUR should be performed prior to committing to conservative therapy. In addition, accurate imaging must demonstrate the absence of extravesical disease, regional lymph node metastases and distant metastases. An early pelvic lymphadenectomy study by Skinner in the days prior to routine preoperative imaging determined an incidence of 5-10% of occult lymph node metastases in patients with pT1 tumors on cystectomy.[6] Subsequent studies have demonstrated that even with MRI imaging, the false negative rate of lymph node staging can be as high as 15%.[7] Newer technologies will hopefully allow us to detect lymph node disease with better sensitivity and allow us to more confidently treat T1G3 disease conservatively.

### The addition of BCG maintenance to TUR prevents recurrences and progression

Managed by TUR alone, T1G3 disease after mean follow-up of 10 years has a 74% recurrence rate, 32.5% progression rate and 50% 10-year survival rate.[8] Certainly, the addition of BCG has improved upon these historical results. Several intravesical studies have consistently demonstrated that BCG administration in addition to standard resection is superior to resection alone in significantly decreasing the number of tumor recurrences.[9] The BCG therapy is also successful in reducing tumor progression when delivered in a maintenance regimen. A recent meta-analysis demonstrated that BCG reduced the odds of tumor progression by 27% (*P* = 0.001) when compared to controls but only in the subgroup of studies that employed maintenance BCG.[10] Unfortunately, extrapolation of data remains a real issue since only 108 patients (2%) of the 5456 patients had G3 tumors.

A study specifically assessing progression in T1G3 disease treated with induction and maintenance BCG found that after a median follow-up of 85 months, 32 (62.7%) of 51 patients remained progression-free, nine (17.6%) progressed, eight (15.7%) died of other causes and two (3.9%) were lost to follow-up.[11] Importantly in this study, disease-specific survival among all patients was 86% which is comparable to that of most radical cystectomy series with T1 disease. The Southwest Oncology Group (SWOG) 8507 trial randomized patients with carcinoma in situ (CIS) or high-risk TCC (Ta or T1 disease with two tumors within one year, ≥3 tumors in most recent six months or CIS on random biopsy) to either BCG induction therapy alone or BCG induction and maintenance therapy. This study demonstrated a decreased median recurrence-free survival in the induction therapy only group versus the induction and maintenance therapy group (36 months versus 77 months, *P* < 0.0001). More importantly, progression was less in the maintenance group as median worsening-free survival was 111.5 months in the no maintenance group and unmeasurable in the maintenance group (*P* = 0.04). However, significant Grade 3 toxicity was noted in one-fourth of the patients receiving maintenance therapy and ultimately only 16% of the maintenance arm actually received all of the planned cycles of treatment.[12] Nevertheless, most patients received at least two cycles of maintenance dosing.

### Several studies confirm high progression-free survival with TUR and BCG

Selected patients with T1G3 disease can be managed conservatively with excellent long-term outcomes. A study from Italy reported a progression rate of 15%, recurrence rate of 33% and overall bladder-cancer specific death rate of 6% in 81 patients with T1G3 tumors treated with TUR and maintenance BCG with median follow-up of 76 months.[13] A recent combined analysis from seven European trials demonstrates that for T1G3 disease, the five-year probabilities for recurrence and progression after TUR are 46% and 17%, respectively. The risk increases with increasing number of tumors, tumor size, prior recurrence rate and concomitant CIS.[14] A multicenter study which analyzed the outcomes of T1G3 disease treated with TUR alone, TUR and BCG and immediate cystectomy revealed that TUR and BCG had the best five-year disease-free survival (80%, *P* = 0.02) among the three treatments. Although the comparison to cystectomy may not have been entirely accurate given that 57% of the cystectomy group actually had muscle-invasive disease on final pathology, TUR and BCG was clearly superior to TUR alone not only in disease-free survival but also in recurrence and progression rates.[15] Overall, the results across several different studies for T1G3 disease managed by TUR and varying regimens of BCG demonstrate recurrence rates of 16-70% and progression rates of 8-33%.[16]

### BCG is superior to other intravesical therapies and has manageable toxicity

None of the other intravesical therapies available have demonstrated any reduction in tumor progression as single agents,[17] but have demonstrated efficacy in decreasing tumor recurrences. Perioperative mitomycin C given within 6 hours of TUR can reduce recurrence by 39% (OR 0.61, P < 0.0001) by theoretically preventing tumor implantation.[18] Several different combinations and sequences of administration of intravesical agents are being evaluated to see if any can offer benefits over BCG alone. Although one of these studies has demonstrated a tumor progression rate as low as 7.4%,[19] none of these studies have convincingly demonstrated any incremental reduction in tumor progression compared to BCG alone. As a result, induction and maintenance BCG therapy is the most effective intravesical agent for preventing progression and is equivalent to other therapies in preventing recurrences in T1G3 disease.

Unfortunately, the toxicity of BCG highlighted in the SWOG 8507 study may compromise the ability to apply maintenance regimens routinely. A more recent study, however, suggests that only 20% of patients find maintenance therapy intolerable leading to discontinuation and inadequate therapy.[20] Dose reduction by 60% in each individual dose has demonstrated no difference in recurrence-free and progression-free survival at five years in patients with T1G3 disease, with the benefit of lower toxicity (Grade 3 or 4 toxicity decreased from 21% to 11%).[21] Further long-term studies are needed to confirm these results, but preliminary evidence suggests that BCG may be given at lower dose, thereby making its risk/benefit ratio more favorable.

### Cystectomy is over-treatment in one-third of patients

For T1G3 disease treated with BCG, cumulative data has resulted in the “rule of thirds” as one-third of the patients will never have a recurrence, one-third of the patients will require a deferred cystectomy and one-third of the patients will ultimately die of metastatic disease.[22] This is obviously an oversimplification but it can at least be said that up to one-third of patients undergoing a radical cystectomy have nonmuscle-invasive disease[23] and probably do not require bladder removal as primary treatment. This might mean that we are not selecting the proper patients for cystectomy. In fact, the bladder can be spared in up to 69% of patients at a median follow-up of 76 months with conservative therapy.[13] Furthermore, outcomes after radical cystectomy for T1 disease at 10 years are variable as some studies demonstrate overall survival as low as 50% and cancer-specific survival as low as 72%.[24] The rates are lower when Grade 3 disease is factored in. Certainly, cystectomy is appropriate for certain patients but cannot be applied indiscriminately to all patients with T1G3 disease.

## CASE FOR CYSTECTOMY

### BCG side-effects and failures

Although life-threatening BCG sepsis is rare (0.4%), most patients (90%) still experience the discomfort of BCG cystitis characterized by dysuria, suprapubic pain and urinary frequency.[25] Bacille Calmette-Guérin (BCG) therapy requires routine urethral catheterization and dwell time within the bladder which hampers a patient's quality of life. A questionnaire-based study found that quality of life was either moderate or bad in 85% patients during initial induction BCG therapy.[26] Maintenance therapy can especially be difficult as only 16% of patients in the SWOG study actually completed all of their treatment doses. Assuming that patients can tolerate therapy, BCG failures can occur in 20-40%[27] of patients and result in intractable disease.

Response to other single-agent intravesical regimens is marginal in patients with BCG failure. In a randomized prospective study comparing the response rates of mitomycin C and BCG in patients with nonmuscle-invasive disease, crossover to the other intravesical therapy was allowed in patients with recurrence and was successful in 39% of patients with second-line BCG but only in 19% of patients with second-line mitomycin C.[28] Similarly, response rates with six-weekly intravesical instillations of valrubicin were only 21% in patients with BCG-refractory CIS.[29] Although modest response rates can occur with second-line intravesical therapy with mitomycin C or valrubicin, BCG and interferon can reduce the recurrence rate to 52% and progression rate to 4.3% in patients with BCG-refractory disease.[30] This combination may become a viable and standard option for second-line treatment in previous BCG failures, but BCG failures are best treated by radical cystectomy for long-term disease-specific survival.[31]

### Significant clinical under-staging

Proponents of early cystectomy argue that patients are considerably under-staged by transurethral resection of tumor and current imaging modalities. As mentioned earlier, the Herr study demonstrated that with repeat TUR of nonmuscle-invasive disease, up-staging can occur in 29% of patients.[5] Ghoneim *et al.*, demonstrated clinical under-staging in 62% of patients originally thought to have cT1 disease but who were up-staged after cystectomy.[32] The majority of patients in these series had squamous cell carcinoma tumors which are relatively less frequent in the United States. A study from the United Kingdom of T1G3 TCC demonstrated up-staging of disease at the time of cystectomy in 55% of patients with multiple T1G3 tumors and/or concomitant CIS. Furthermore, none of these 17 patients had pT0 at the time of cystectomy.[33] North American series cite a 30%[34] to 40%[35] incidence of under-staging in patients with cT1 disease. In fact, pathologic up-staging to muscle-invasive or metastatic disease was significantly associated with decreased survival (*P* = 0.042).[35] Radical cystectomy in these patients appears to be reasonable as it not only addresses under-staging of the primary tumor but also addresses under-staging of the lymph nodes that may result from existing imaging studies. Furthermore, with accurate pathologic assessment of the primary tumor and regional lymph nodes, patients appropriate for adjuvant chemotherapy can be identified. Finally, if muscle-invasive disease is present, radical cystectomy provides for aggressive and potentially curative therapy.

### BCG delays progression but does not prevent it

It is unclear whether BCG actually prevents progression or just delays progression as follow-up is limited in most series. Indeed, rates of progression appear to be greater in studies with longer follow-up. A study at Memorial Sloan-Kettering Cancer Center followed 86 patients with high-risk disease treated only with transurethral resection (TUR) and BCG for 15 years. By the time of analysis, 53% of the patients progressed with 36% ultimately requiring cystectomy with projected 15-year disease-specific survival of 63%.[36] Similarly, overall rates of recurrence also appear to be greater with time. Shahin and colleagues demonstrated disease recurrence in 70% of patients treated with TUR and BCG after a median follow-up of 5.3 years. Over 10 years, 30% of all patients in the study died of disease progression.[22] As a result, delayed progression may lead to a gradual decline in survival because when a delayed or deferred cystectomy is finally performed, the window of opportunity for long-term survival may have been missed.

### Early versus delayed cystectomy

Survival outcomes are better with earlier cystectomy. A classic report from Germany compared the results of early versus delayed cystectomy for T1 disease and found better five-year recurrence-free survival in the early group (90% *vs.* 61.5%).[37] Unfortunately, the patients were not randomized and a selection bias for more aggressive disease existed in the delayed group as they were only treated with cystectomy after failing TUR and surveillance. Nevertheless, more contemporary series have reached the same conclusion. A study by Herr and Sogani demonstrated that survival was better with earlier cystectomy (within two years of initial BCG therapy) than delayed cystectomy (more than two years after initial BCG therapy) in patients with nonmuscle-invasive disease that failed BCG (92% *vs.* 56%, *P* = 0.03).[38] In a series of patients from the University of Southern California, five-year recurrence-free survival and five-year overall survival for pT1 disease treated by radical cystectomy was 83% and 72%, respectively.[39] In muscle-invasive disease, TCC is so potentially aggressive that delays greater than three months from the time of diagnosis until surgery can result in more advanced disease and decreased survival.[40] Likewise, this concept may apply to T1G3 lesions as they transition from T1a disease (lamina propria invasion above muscularis mucosae) to T1b disease (lamina propria invasion into muscularis mucosae) to T2 disease.

### Minimal mortality and morbidity with cystectomy

The perioperative mortality often cited with contemporary cystectomy series is approximately 3% and does not appear to be related to the choice of urinary diversion performed. Early complications can occur in up to 28% of patients and most can be managed without additional surgery.[39] Quality of life in bladder cancer patients after radical cystectomy and orthotopic bladder substitution is similar to quality of life of a normal matched population in terms of overall quality of life. A study demonstrated no difference in the prevalence of depression, anxiety, subjective quality of life and low or moderate psychological well-being between the two groups.[41] Furthermore, quality of life appears to be equivalent among different types of urinary diversion.[42] Potency is partially age-dependent but can be preserved in up to 20-62% of patients through nerve-sparing techniques.[43]

## PREDICTORS OF DISEASE PROGRESSION

Making the difficult decision to perform a cystectomy is currently guided by the details of the pathology report and certain patient factors. However, attempts to identify and validate certain markers predictive of progression may enable us to better predict which patients with T1G3 disease are likely to progress and therefore candidates for immediate cystectomy. The following is a review of these markers.

### Chromosomal alterations

Altered protein expression of p53, p21 and pRb by immunohistochemistry in archived radical cystectomy specimens correlates with increased time to recurrence, decreased survival and increased recurrence rate.[44] The value of p53 expression in predicting recurrence and progression in T1G3 disease, however, has mixed results. In patients with pT1 tumors, five-year recurrence rates were 7% in the absence of p53 nuclear reactivity and 62% in the presence of p53 nuclear reactivity.[45] In 26 patients with high-risk nonmuscle-invasive TCC (24 with T1G3 and two with CIS), the presence of p53 mutations were predictive of response to BCG (72% with mutations failed vs. 38% without mutations, *P* = 0.0075).[46] Some series, however, have demonstrated the inability of p53 status in predicting response to BCG therapy in T1G3 disease.[47,48] At MD Anderson Cancer Center (MDACC), we are currently participating in a Phase I, multi-center clinical trial evaluating the predictive value of urinary microsatellite analysis of chromosomal alterations.

### Molecular markers

Epidermal growth factor receptor (EGFR) can be over-expressed in bladder cancer. A study has shown that EGFR positivity on transurethral specimen of nonmuscle-invasive disease is associated with stage progression (*P* = 0.0004). In the subset of patients with T1G3 bladder cancer, EGFR status was 80% sensitive and 93% specific in predicting stage progression.[49] Cell cycle regulators, by regulating the transition from the G1 to S phase in the cell cycle, also have prognostic significance in T1G3 disease. On mutltivariate analysis, over-expression of three of these regulators, cyclin D1, cyclin D3 and p53, was predictive of progression-free survival suggesting that cell cycle inhibitors may have therapeutic importance in T1G3 disease.[50]

### Pathologic risk factors

The presence of CIS increases the risk of progression in T1G3 disease treated with TUR and BCG. In a nonrandomized study of patients treated with TUR and single six-week BCG induction therapy, five-year progression rates were 49% for patients with pT1 tumors and CIS *vs.* 20% for patients with CIS alone (log-rank test *P* = 0.013).[51] The rates may have been lower had a maintenance regimen of BCG been employed. On multivariate analysis, both CIS and tumor size ≥3 cm are predictive of progression in T1G3 disease treated with induction and maintenance BCG. On univariate analysis, recurrent tumors, solid tumors and early recurrence with T1G3 disease (after first induction course of BCG therapy) were significantly associated with progression.[11] The lack of response to three months of intravesical therapy has been found to be predictive of progression in T1G3 disease.[52] Certainly, the presence of any variant histology such as micropapillary, small-cell, sarcomatoid and nested histology is evident of more aggressive disease that may require chemotherapy and/or radical cystectomy.[16] Additionally, the presence of multi-focal T1G3 tumors and tumor in the prostatic urethra may be prognostic of up-staging as well. Finally, the presence of lymphovascular invasion (LVI) in either the TUR specimen or radical cystectomy specimen is significantly associated with decreased bladder cancer specific survival (*P* = 0.15).[35]

Several reports have emerged suggesting that sub-classification of the depth of T1 invasion may provide excellent prognostic information. By classifying T1 disease into T1a (invasion above muscularis mucosae) or T1b (invasion into muscularis mucosae), one report demonstrated that 6.7% of T1a tumors progress *vs.* 53.5% of T1b lesions (*P* < 0.01) regardless of size, tumor grade and number of tumors.[53] Other groups have confirmed the prognostic significance of sub-staging T1 disease and demonstrate that it is possible in up to 87% of cases.[54] However, even at a large national meeting (Society of Urologic Oncology meeting, December 2006), it was noted that most pathologists do not routinely report the level of lamina propria invasion since it may be too ‘operator-dependent’. However, if able to accurately determine the depth of invasion, this may be a reliable prognostic factor.

## CONCLUSION

In patients undergoing conservative therapy for T1G3 disease, the evidence suggests the need for a repeat TUR and appropriate imaging to ensure that the tumor is adequately staged. If pathology demonstrates T1G3 disease in the absence of other high-risk features (noted above), intravesical therapy with induction and maintenance BCG are essential to decrease the risk of recurrence and progression. The use of maintenance therapy varies across published reports and practices, but we adhere to the “6 + 3” BCG regimen outlined in the SWOG 8507 study (also known as the “Lamm regimen”). The patient needs to be counseled on a roughly 30% probability of no progression or recurrence with the 30% risk of requiring a cystectomy due to progression of disease before committing to conservative therapy. Conversely, the patient should consider that immediate cystectomy can result in five-year disease-specific survival greater than 90% but at the expense of living with a urinary diversion.

Patient factors may ultimately drive this decision as the presence of immunosuppression or previous BCG toxicity may make conservative therapy infeasible while the presence of advanced age or poor surgical candidacy may make cystectomy too risky. In most patients, we are currently driven by certain pathologic features in determining which therapeutic path to embark upon. The presence of CIS, large tumors and multi-focal disease start to tip the balance in favor of cystectomy but certainly BCG induction and maintenance therapy is still reasonable. Early recurrence or progression of disease on BCG (within three months), LVI, inability to treat completely with TUR, T1b disease and variant histology are reasonable criteria to change from conservative therapy to radical cystectomy. In patients with recurrence of low-grade disease or in poor surgical candidates, repeat BCG induction or BCG and interferon appear to be reasonable strategies but require aggressive surveillance.

The treatment of bladder cancer potentially hampers quality of life and imposes morbidity upon patients. The management of T1G3 disease mandates periodic cystoscopic evaluation and urethral catheterization to evaluate and treat recurrences, respectively. Nevertheless, disease progression can occur and even with cystectomy, surveillance is still essential. With the various treatments and surveillance, it comes as no surprise that bladder cancer is the most expensive cancer to treat from diagnosis to death with costs ranging from $57,629 (based on 1995 values)[55] to $187,241 (based on 2001 values)[56] per patient. However, the average cost of a radical cystectomy alone can be as high as $55,975 per patient.[57] The sobering economic realities of this disease not only remind us of the persistence and aggressiveness of this malignancy but also of our inability to develop effective therapies against this cancer to prevent recurrence and progression of disease.

More concerning than the economic realities are the poor outcomes in patients with progression of disease identified late so that the disease is no longer organ-confined at the time of cystectomy. The five-year overall survival rate drops from 78% in organ-confined disease to 47% with extravesical disease.[39] The rates are even lower in the presence of positive lymph nodes. Clearly, we need to identify which T1G3 patients' progress and to identify them prior to initiating therapy. Furthermore, the need for centralized pathologic review cannot be stressed enough as the course of treatment hinges on accurate pathologic diagnosis. At MD Anderson Cancer Center, we are investigating the ability of urinary markers to identify patients with bladder cancer. The ultimate future may lie in the molecular sub-typing of T1G3 tumors and determining relative levels of cell cycle regulators, growth factor receptor expression and chromosomal alterations. Hopefully, further research into this field will result in molecular stratification of patients that can accurately predict the likelihood of progressive disease. Through such an analysis, we may be able to incorporate a patient's pathologic features, urinary markers and molecular sub-typing into a nomogram and accurately determine which patients require a cystectomy and which patients do not. Of course, the benefits of such an approach must be balanced against the availability, processing time and costs of such individualized patient testing.
